# Developing a Dementia Research Registry: a descriptive case study from North Thames DeNDRoN and the EVIDEM programme

**DOI:** 10.1186/1471-2288-11-9

**Published:** 2011-01-27

**Authors:** Steve Iliffe, Lisa Curry, Kalpa Kharicha, Greta Rait, Jane Wilcock, David Lowery, Archana Tapuria, Dipak Kalra, Craig Ritchie

**Affiliations:** 1 Research Department of Primary Care & Population Health, University College London, Royal Free campus, Rowland Hill St., London NW3 2PF, UK; 2 West London Mental Health Trust, London, UK; 3 Central & NW London NHS Foundation Trust, London UK; 4 Centre for Health Informatics and Multiprofessional Education University College London Holborn Union Building, Highgate Hill, London N19 5LW, London UK; 5 West London NHS Mental Health Trust, London UK

## Abstract

**Aim:**

To describe the development of a dementia research registry, outlining the conceptual, practical and ethical challenges, and to report initial experiences of recruiting people with dementia to it from primary and secondary care.

**Background:**

Women, the oldest old and ethnic minorities have been under-represented in clinical trials in dementia. Such under-representation biases estimates of absolute effect, absolute harm and cost-effectiveness. Research on dementia should include patient populations that more exactly reflect the population at risk. One of the impediments to this is the lack of a suitable tool for identification of patients suitable for studies.

**Construction & contents:**

A technology development methodology was used to develop a registry of people with dementia and their carers. This involved phases of modelling and prototype creation, 'bench testing' the prototype with experts and then 'field testing' the refined prototype in exemplar sites. The evaluation of the field testing described here is based on a case study methodology.

**Utility:**

This case study suggests that construction and population of a dementia research registry is feasible, but initial development is complex because of the ethical and organisational difficulties. Recruitment from primary care is particularly costly in terms of staff time and only identifies a very small number of people with dementia who were not already known to specialist services. Recruiting people with dementia through secondary care is a resource intensive process that takes up to six months to complete. Identifying the components of a minimum dataset was easy but its usefulness for pre-screening potential research populations has yet to be established. Acceptance rates are very high in the first clinic to recruit to the registry, but this may reflect the efforts of registry 'champions'.

**Discussion and Conclusions:**

Easier recruitment may perpetuate potential selection biases and we are not yet able to assess the representativeness of the research-ready population recruited to the registry. The need to recruit from wider populations, through primary and social care, remains. The success of this registry will be measured by the proportion of people from it who are recruited to research projects, and its impact on overall accrual to studies.

## Background

UK government policy is to maintain people with dementia syndromes in their own homes for as long as possible [1]. However, the needs of people with dementia and their carers' are inadequately addressed at all key points in the illness trajectory, from diagnosis through to end of life care [2]. Further research is required to address the obstacles to the timely recognition of dementia syndromes in primary care [3], the support for people with dementia and their families after diagnosis, carer strain, what factors predict the transfer of people with dementia syndromes to institutional care, interventions to manage incontinence and challenging symptoms [4] and the therapeutic options available to clinicians which are currently sparse and insufficiently evaluated [5].

There are some treatments for people with Alzheimer's disease shown in trials to be effective in modifying symptoms [6] and emerging therapies will need rigorous evaluation in large-scale trials. The next target for chemotherapeutic approaches in Alzheimer's disease is the development of disease-modifying drugs, but the design of these trials raises many questions. Which populations should be studied, for how long and with which principal and secondary endpoints? [7].

These questions may be difficult to answer. Difficulties in ensuring that samples are representative have meant that people with dementia included in clinical research have been systematically younger than the general population of people with dementia and that women, the oldest old and ethnic minorities have been under-represented. Such under-representation may not always affect the external validity of relative effect estimates, but measures of absolute effectiveness, absolute harm and cost-effectiveness are associated with underlying risk levels in different socio-demographic groups and current under-representation will bias absolute effect estimates [8]. Research on dementia could gain much from the study of patient populations that more appropriately reflect the population at risk [9]. This age differential is significant given the potential delays in dementia diagnosis, progression of dementia and diminishing capacity to give informed consent to participate in a study.

Primary care-led studies could in theory address these methodological problems because of the heterogeneity of the community population and given the growing expertise in trial design and implementation, but in practice we know from recent trials that recruitment to studies on dementia through general practice is problematic [10,11].

### Promoting dementia research

These difficulties in recruitment to dementia research prompted the National Institute of Health Research to establish the Dementia and Neurodegenerative Research Network (DeNDRoN). The Dementia and Neurodegenerative Research Network aims to improve the speed, quality, and integration of research in dementias and other neurodegenerative diseases, resulting in improvements in prevention, diagnosis, treatment and care for patients. DeNDRoN facilitates the development, conduct and delivery of clinical trials and other well-designed studies by:

1. Coordinating focused, effective investment in National Health Service (NHS) research infrastructure to ensure that quality research, funded by both commercial and non-commercial organizations, becomes embedded in clinical practice.

2. Building on the research strengths already present in the UK as well as increasing general research capacity in the field of dementia and neurodegeneration.

3. Promoting collaboration between patients, carers, researchers, clinicians, academics, NHS Trusts, funders and industry, to enhance sharing of resources and expertise.

One of the impediments in accomplishing clinical trials for the treatment of dementia is the lack of a suitable tool that would facilitate identification of patients who might be recruited for studies. Registries for patients with Motor Neurone disease and Huntington's disease have been long established but to date there is no equivalent registry for such a large patient group, those with dementia syndrome and earlier clinical, cognitive manifestations of neurodegenerative disease in the UK. The problems of recruiting the appropriate populations to trials prompted the DeNDRoN to test the concept of a research registry of people with dementia and cognitive impairment (presumed secondary to neurodegeneration) who would express an interest in participating in dementia and cognitive impairment research in general, rather than specific studies.

### Research registries

There is now considerable experience of developing research registries, particularly in North America. Many registries have been developed to facilitate epidemiological studies [12] but can also offer an organized and systematic way to maintain contact with participants from previous research and recruit an even more diverse pool of subjects interested in participating in future studies [13].

However, there are difficulties in developing research registries. Registries have been used in dementia research, to study the clinical expression of Alzheimer's disease [14] and to improve the flow of information in order to increase research participation [15]. The US Consortium to Establish a Registry for Alzheimer's disease (CERAD) [16] has functioned as a vehicle for a wide range of studies and as a mechanism for developing and testing dementia-specific instruments. In 2008 the Leon Thal Symposium proposed the development of a US National Registry and Database to meet the multiple needs of the research field, including the development of a a research programme on prevention [17]. Similarly, the European Alzheimer's Disease Consortium in 2010 proposed the construction of international research registries for studies of familial Alzheimer's disease and for therapeutic trials [18].

Whilst registries based on routinely collected data can offer opportunities for research they pose problems of data organisation and accuracy for researchers [19]. Prospective collection of additional data requires organised outreach from the Registry to patients, providers and staff, integration of the registry into pre-existing clinical routines and addition of reminder systems to workstations [20]. Unique challenges in recruiting and retaining participants with neurological disorders for research studies include cognitive deficits in participants and the complex ways in which many neurological conditions present [21].

The perceived advantages of a research registry were that providing an opportunity for patients to show their interest in research could allow pre-screening of research- ready populations for different types of study, allow more accurate assessments of study feasibility (because the potential research population would be known), and create the basis for cohort studies. This paper describes a project to establish a dementia registry and reports initial experiences of recruiting people with dementia through primary and secondary care.

## Construction and Contents

### Evaluation

The evaluation of the field testing described here is based on a case study methodology. Case study methods are appropriate when investigators desire or are forced by circumstances to define research topics broadly, to cover contextual or complex multivariate conditions and to rely on multiple sources of evidence [22]. The all-encompassing feature of a case study is its intense focus on a single phenomenon within its real-life context [23]. The research questions in this case study of the dementia research registry are:

1) Is it feasible to develop and sustain a research register for people with dementia?

2) What are the actions and resources required to develop and implement a dementia research registry?

3) What are the clinical data capture requirements for a dementia registry for the purpose of clinical trials recruitment?

4) What are the likely recruitment rates to a dementia research registry?

### Stakeholders

DeNDRoN is funded by the UK National Institute of Health research (NIHR) and has specific objectives that include increasing the number of principal investigators, research sites and numbers of patients recruited to trials. North Thames DeNDRoN is one of the seven regional DeNDRoN networks and is a collaboration between three universities [Imperial College London (ICL), Queen Mary's University of London (QMUL) and University College London (UCL)] and 36 NHS Trusts covering all North London Boroughs plus areas of Essex, Hertfordshire and Bedfordshire (26 Acute Trusts, 10 Mental Health Trusts). It is hosted by one NHS Trust.

The EVIDEM programme (Evidence-based Interventions in Dementia) funded by the English National Institute of Health Research also proposed to create a cohort of up to 2000 people with dementia and their carers, as the basis for recruiting individuals to studies on early diagnosis, continence management, management of behavioural and psychological symptoms in dementia, end-of-life care and the application of new legislation about giving and obtaining consent (the Mental Capacity Act 2005).

### Registry design

The development of the registry was based on a standard technology development methodology, originally derived from the construction of decision support systems [24]. This involves phases of modelling and prototype creation, 'bench testing' and refining of the prototype with experts and then 'field testing' of the refined prototype in exemplar sites.

A co-design approach [25] was taken, bringing together researchers (in the EVIDEM programme), research network developers (in DeNDRoN) and people with dementia and carers, through the Patient and Public Involvement arm of DeNDRoN.

The design team met on six occasions during 2008-9 and held monthly teleconferences to review progress. The design team consisted of five members from the EVIDEM programme (SI, DL, GR, JW and KK) and two from North Thames DeNDRoN (CWR, LC) bringing together academic, clinical and research network expertise. The design team developed a prototype registry, 'bench tested' it with other experts in the field, and then initiated recruitment to it, initially in one specialist pilot site but also in selected general practices.

Expert advisors from the Centre for Health Informatics at University College London were recruited to the design team to develop the electronic database for the registry (AT, DK). The proposals for the registry were discussed with DeNDRoN's patient and public involvement working group (made up of DeNDRoN regional workers and members of third sector organisations) and Forum (made up of people with neurodegenerative diseases and their carers).

### Modelling, 'bench testing' and prototype development

The objectives agreed by the design team were:

a) To identify people with dementia and their carers through primary and secondary health care, social care, community care and voluntary sector organisations in the North Thames DeNDRoN region.

b) To invite patients to join a research registry.

c) To gain consent for a minimum dataset of information about patients to be held on the research registry.

d) To enable clinical research staff and registered research staff to search for patients relevant to a set of user-defined parameters, and then use that retrieval set as the basis for making contact (through the patients' clinicians).

e) To enable the registry staff to maintain a list of studies to which the patient has been invited, is deciding about, has consented too or is participating in.

f) To enable appropriate matching of registry members to research projects and further anonymised analyses.

g) To manage all such data securely, using role based access and maintaining an audit log.

Recruitment to the Registry would occur in the geographical area covered by the North Thames DeNDRoN. Recruitment would occur through primary care, secondary care, social care (e.g. care homes), community care (e.g. community nursing services, Admiral Nurses) and third sector (voluntary) organisations (e.g. Alzheimer's Society).

The target population was defined as people of any age with any form of dementia residing in the community or residential care within the defined geographical area.

The inclusion criteria chosen were: People with either a formal specialist (imaging/neuropsychological) or informal generalist diagnosis of dementia as well as participants with cognitive impairment presumed secondary to an underlying neurodegenerative disease. The case definition includes different types of dementia syndrome and people with dementia of differing severity (from early to late dementia as well as the - much debated - Mild Cognitive Impairment). We agreed to include non ICD10-DSM-IV diagnoses, but the source and quality of the diagnosis would be a field within the registry to allow prospective filtering to match the quality needs of later research projects. The exclusion criteria chosen were: 1) People who do not speak English for whom an interpreter could not be located and 2) those whom their clinician believed it would be inappropriate to approach, for specific reasons like receiving end-of-life care, treatment for severe co-morbidity, or major behaviour disturbance.

### Ethics committee approval

Although the primary aim of the registry was to support research, the design team felt that it was essential to seek ethics committee approval, in part because diminishing capacity to consent to participation in research is a feature of dementia syndrome. In addition the Data Protection Act requires that all patients who are identified for research projects have given their consent to be identified in this way, and the design team believed that an ethics committee would provide another layer of expert opinion about how best to explain the purpose of the registry. Finally, the rigorous and well documented consenting process that has to be followed as per the granting of the ethics approval provides a clear and auditable pathway from dissemination of information regarding the registry through the information sheets for patients and carers, the assessment of capacity and the storage of critical documents to defend against future challenges which may arise.

It was also clear that recruitment of large numbers of people with dementia and their carers would require Research Management and Governance approvals across multiple sites and sectors, and information management approvals for data storage. In addition, the team had to develop a minimum dataset and database, and devise mechanisms for capturing data in primary and secondary care, and through other routes like care homes and third sector organisations.

### Constructing the minimum dataset

A minimum set was developed in three stages. In the first stage written commentary on the secondary care requirements of the dataset were gathered from the North Thames DeNDRoN's executive board, supplemented by individual discussions with researchers within the local network. In the second stage face-to-face interviews with primary care clinicians were conducted to discuss the potential for using data from the General Practice reimbursement mechanism (the 'Quality & Outcomes Framework') for dementia. In the third stage members of North Thames DeNDRoN gave feedback on the minimal dataset fields generated in the previous two stages and the dataset was refined based on this feedback. Table 1 shows the contents and data fields of the minimum dataset.

**Table 1:** The minimum dataset

For all practices we will record	Location (Primary Care Trust -PCT)
	
	**Deprivation index score**.
For all clinics we will record	Specialist
	Clinic location

For other services we will record	Location e.g. Nursing Home, Supported Accommodation, Elderly Mentally Infirm (EMI) home
	
	Key worker details

For all participants in the registry the following information (extracted from practice or clinic notes) is recorded where possible:	Demographic details (name, date of birth, gender, marital status, first language, ethnicity, address, postcode, housing status, National Health Service number)
	
	Carer information (name, date of birth, gender, address, postcode, relationship to person with dementia)
	
	Practice details (name, address)
	
	Specialist details (name, clinic details)
	
	Cognitive status (date of most recent test and score)
	
	Functional status (date of most recent test and score)
	
	Behavioural/Neuropsychiatric status
	
	Investigations (imaging and dates)
	
	Specific dementia medication
	
	Co-morbidity (e.g. depression, CVD, diabetes)
	
	History of participation in trials/studies

We were aware that information recorded in notes would be variable across services and sites. This minimum dataset was based on data known to be routinely collected in secondary care clinics assessing patients with cognitive disorders and to a lesser extent in primary care (for example, functional status data may not be routinely recorded in primary care notes). The design group intended that the minimum dataset would evolve over time to be consistent amongst collaborating centres, as far as pragmatically possible.

### Confidentiality

A unique identifier is assigned to all participants on the registry, so that data are held anonymously. A file linking name and unique identifier is stored separately and securely and in accordance with the Data Protection Act. This will be held until the participant indicates that s/he no longer wishes their data to be included on the registry, and six monthly reviews will allow reaffirmation of registry status. Six monthly reviews were chosen because of the relatively rapid health status changes that can occur in dementia syndrome. It is intended for the registry to be comprehensive and to be able to include all patients seen in the North Thames DeNDRoN region in order to be representative of the patient population.

### Duplication

Information on whether the patient has been/or is currently participating in research studies will be included on the registry to avoid patients being approached for participation in multiple projects and registry managers will cross check key identifiers (name, date of birth, NHS number) of potential new participants to ensure that people and their carers are not approached repeatedly.

### Access

National and regional researchers wanting to access the Registry will need to approach North Thames DeNDRoN in the first instance. Prioritisation of studies within the North Thames DeNDRoN portfolio by local researchers is anticipated and access to the registry will reflect this prioritisation. Governance of the Registry will be managed through DeNDRoN's national co-ordinating centre.

## Utility

Physical construction of the research registry and its use at the first sites provided experience of the practical problems involved in recruiting people with dementia to a research registry. These included identification and invitation of potential participants, judgement of capacity, and obtaining both official permission and actual support from practitioners and administrators to recruit through NHS services.

**Figure 1:**
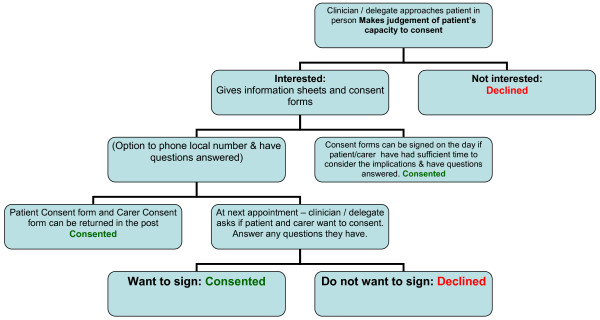
**Recruiting patients and carers to the Dementia Registry**.

**Identification of people with dementia **from medical records complied with recommendations from the Patient Information Advisory Group (PIAG). These recommendations allow only members of the patient's usual clinical care team to pre-screen patient notes to identify those suitable for the registry. The lead clinician (or other member of the normal clinical team responsible for the patient's care) would then make the first contact with the patients identified, either in a face-to-face meeting or by letter or telephone. This contact would be only to inform the individual or their family about the registry; enrolment would usually take place separately from the clinical encounter in which the information about the registry was given. There are exceptions to this, as some people are keen to enrol immediately rather than wait until their next appointment. In cases where people feel they have had sufficient time to consider their decision, their consent can be taken on the same day as they receive the information. Figure 1 shows the recruitment path and steps for an individual enrolled through a memory clinic. This process is likely to vary slightly to reflect differing care pathways in different memory clinic services.

### Judgement of Capacity

To ensure that people at all stages of the disease process were able to join the registry required judgements about capacity. (This proved particularly difficult in primary care - see below). A recent analysis of ability to consent to research in a therapeutic trial of Gingko Biloba found approximately 70% of individuals with mild-moderate dementia could not give valid consent to research participation [26]. In the case of individuals who are not able to give informed consent, UK Medical Research Council and European Union-Good Clinical Practice guidelines and the principles of the UK Mental Capacity Act 2005 were followed, and assent sought from a relevant consultee. This is also in accord with internationally accepted guidelines on research involving human subjects with dementia [27].

### Seeking Permissions

Research Management and Governance offices at each NHS Trust were approached for permission to approach Trust staff and to engage them in the development of the database. Seeking multiple permissions across provider organisations in primary and secondary care proved to be a lengthy process, taking up to five months. This process was not made easier by high staff turnover rates in the DeNDRoN research network itself and the time needed for training, Criminal Research Bureau checks and research passport applications for new study officers. The steps required to engage a new clinical site as a recruitment site for the registry are summed up in Table 2.

**Table 2:** Getting a new specialist site to recruitment stage requires:

A principal investigator to act as champion for that site
Local Research Management & Governance approval

Resources for a local co-ordinator, who initially carries out and then coordinates data entry, acts as contact person for data queries and liaises with site staff about recruiting patients. To date the financial resources have come from different streams of research network funding. New staff may need to be recruited, or honorary contracts established for those already in other posts.

Agreement from local IT departments who need to give new staff access to electronic databases, and to set up shared drives where none existed previously. The local information governance manager needs to be satisfied about data security.

Service manager agreement to provide office space, promote the use of the registry to front line clinical staff, allow computer use and staff involvement in seeking consent, as well as facilitating best working practices for each site.

Site team involvement in supporting the lead clinician in identification of patients to inform about the study

Recruitment of patients and carers to the research registry generated important lessons about sites of recruitment, and about data governance.

### Recruitment in secondary care

**Figure 2:**
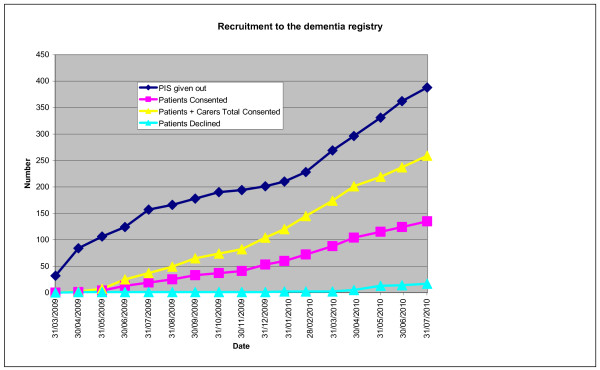
**Recruitment to the registry 2009-2010**.

Recruitment began in the first Mental Health Trust in early March 2009, and three other Trusts began patient recruitment in the next 12 months, with four more initiating involvement. Figure 2 shows the rates of invitation and recruitment to the registry, the numbers engaged through the registry in trials, over a one year period. Acceptance of the invitation was high, at over 90%, but the rate of recruitment has been determined by the pattern of clinic attendances, with a gap of three to six months between the invitation to join the registry and acceptance.

### Recruitment in primary care

Tests of recruitment began with five practices that were already part of the EVIDEM programme in autumn 2009. Of 72 people with dementia identified from these five general practices and sent information by post about the research registry, three responded that they were not interested in research or finding out more and 18 responded that they were interested and want to know more. Fifty one did not respond to the invitation.

Amongst those expressing interest in the research registry ten people were attending specialist clinics already working with the research registry, and their details were passed onto the appropriate clinic. Three lived in Care Homes, and an assistant psychologist obtained the carer's agreement to gather data for one of them. She did not meet the patient to assess capacity, but both the GP and the Care Home manager judged that the resident lacked capacity for this decision. The data gathered did not contain information about medication or MMSE score, and the psychologist extracting the data was unsure which of two documented diagnoses was correct. For the other two individuals the assistant psychologist had to meet with distant relatives as care home staff did not feel able to give an opinion about participation in research.

The assistant psychologist also arranged a meeting, at a hospital site, with two of the three who were not seen in any other service. The remaining person not seen in any other service lived in an area distant from any service that the psychologist could invite them to, and the assistant psychologist was uncomfortable with home visits, so this individual's expression of interest was not pursued.

Testing the process of recruitment in general practice was undertaken by the EVIDEM team and in specialist clinics by NT DeNDRoN, but responsibility for subsequent six monthly follow-up and review did not clearly belong to either party and had to be decided through discussion. The situation was complicated by the organisation of research infrastructure in England, where three research networks may be involved in dementia research: DeNDRoN, the Primary Care Research network (PCRN), which recruits general practitioners for research projects, and the Comprehensive Local Research Network (CLRN), which funds the involvement of practitioners in research work. These three types of network do not have the same boundaries, so trilateral negotiations were necessary in different geographical locations to allow the EVIDEM team to test recruitment to the registry through general practice.

### Data governance

A decision had to be made about who would ultimately hold and be responsible for the data collected in this way. The information sheets state that documents and registry data would be stored by the registry team at North Thames DeNDRoN. However, North Thames DeNDRoN is not a legal entity. As such, documents and registry data are stored by West London Mental Health Trust in accordance with agreements from North Thames DeNDRoN for the prototype, and are only accessible by the clinical and research staff within the involved NHS Trust's boundaries.

## Discussion

### Feasibility

The North Thames DeNDRoN dementia registry is a pioneering project in the United Kingdom. This case study suggests that construction and population of a dementia research registry is feasible, but that the initial development is complex because of the ethical difficulties in dementia research and the organisational difficulties in embedding research projects in NHS clinical services. Recruitment from primary care has proved problematic; enrolment of patients is particularly costly in terms of staff time especially given the very small number of people with dementia identified who were not already known to specialist services. The logistics of recruitment in memory clinics was relatively easy to establish because of the concentration of patients and staff as well as the rigorous application of care pathways into which the recruitment process can be embedded. Even then, given the timescale of clinic attendance and the restrictions on obtaining informed consent, the recruitment process may take up to six months.

### Resource issues

Recruiting people with dementia to the registry through secondary care is still a resource intensive process. Potential registry members need to be identified as suitable, informed about the registry, met again to obtain consent and to capture information for the minimum dataset, and reviewed every six months to confirm their continued interest and update their dataset. The preliminary steps in gaining the necessary permissions and resources to establish the registry at a new site require effort (a manager able to devote sufficient time) and up to five months of preparatory work (decreasing as time and experience informs the process). However, early investment of effort will ensure that not only will local clinical teams be invested in the process but it also ensures that data collected thereafter will be both accurate and complete. The costs of developing and running the registry are core service support costs and will therefore be borne by the NIHR Clinical Research Network. DeNDRoN proposes to fund the registry through existing funds, given the current financial climate, but it is also part of DeNDRoN's five year strategy (2010-2015) to develop a broad coalition to secure appropriate funding arrangements for the registry in the longer term.

### Clinical Data requirements

Identifying the components of a minimum dataset was an early achievement of the design team, which is being tested as researchers begin to use the register to recruit to studies. The effectiveness of the registry's minimum dataset (as currently designed) as a device for pre-screening potential research populations has yet to be established.

### Recruitment

Acceptance rates are very high in the first clinic to recruit to the registry, but this may reflect the efforts of registry 'champions'. We are monitoring recruitment in more recently recruited clinics where there may be less ownership and hence less commitment to registry development; this will give us an estimate of the likely growth of a dementia research registry within usual NHS clinical practice. Easier recruitment may perpetuate potential selection biases and we are not yet able to assess the representativeness of the research-ready population recruited to the registry; this is an issue that needs to be revisited in the next stages of registry development. The design team will need to reconsider ways of increasing recruitment through primary care, and through care homes and social services, to offset biases inherent in clinic recruitment.

## Conclusions

We believe that the registry will assist in connecting people with dementia and their carers with high quality research studies that will help us answer important questions regarding the pathology, clinical pathways, aetiology, experiences of and best treatments for neurodegenerative disease. Only through careful scrutiny of our processes in developing the registry and articulating the problems faced will we deliver within the DeNDRoN research network a state-of-the-art recruitment tool.

The primary obstacle to the development of the registry has been the complexity of permission processes within the NHS, an experience noted by others [28]. This may change as the registry is adopted by research sites that are not part of the designer group; there staff attitudes and clinical priorities may become more salient, as found in other registries [18]. A number of important characteristics of the registry are awaiting evaluation, including the utility to researchers of the minimum dataset, the representativeness of the population recruited to the registry and the cost per person recruited to studies through the registry. Recruitment from sources other than specialist clinics needs further investigation, to achieve a more representative population of research-ready people with dementia.

The success of this prototype will be measured by the proportion of people from the registry who participate in research studies and the impact that the registry has on overall accrual to portfolio studies. If these outcomes are positive, and if recruitment to the registry becomes part of the activity of other research sites within North Thames, the methodology of the registry will be made available for all local research networks within DeNDRoN.

## Conflicts of interest

The authors declare that they have no competing interests.

## Contributions of authors

CR was one of original designers of the registry, contributed to protocol writing, and submission of ethics application, and is chair of the registry steering group. SI was one of original designers of the registry, contributed to protocol writing and submission of the ethics application, and is CI for the EVIDEM programme. JW is a member of the registry steering group, piloted the minimum dataset and recruitment of primary care practices, is a member of NT Dendron steering group and also Academic Research Manager for the EVIDEM programme http://www.evidem.org.uk

GR is a member of the EVIDEM cohort team, contributed to protocol writing and the ethics application, and is Primary Care Lead for North Thames DeNDRoN. DL is the Trust-based project manager for EVIDEM, and has contributed to the registry's implementation. KK is a member of the registry steering group and Academic Research Manager for the EVIDEM programme http://www.evidem.org.uk. DKprovided IT support for the registry's development, as did AT. LC is the Dementia Registry manager.

All authors have read and approved the final manuscript.

## Pre-publication history

The pre-publication history for this paper can be accessed here:

http://www.biomedcentral.com/1471-2288/11/9/prepub
